# Optimizing Ammonium Polyphosphate–Acrylic Intumescent Coatings with Sustainable Fillers for Naval Fire Safety

**DOI:** 10.3390/ma17215222

**Published:** 2024-10-26

**Authors:** Elpida Piperopoulos, Giuseppe Scionti, Mario Atria, Luigi Calabrese, Antonino Valenza, Edoardo Proverbio

**Affiliations:** 1 Engineering Department, University of Messina, Contrada di Dio-Sant’Agata, 98166 Messina, Italy; giuseppe.scionti@studenti.unime.it (G.S.); lcalabrese@unime.it (L.C.); eproverbio@unime.it (E.P.); 2 Colorificio Atria, Contrada Camarro Formeca, 91028 Partanna, Italy; amario@atria.it; 3 Engineering Department, University of Palermo, Viale delle Scienze, 90128 Palermo, Italy; antonino.valenza@unipa.it

**Keywords:** intumescent coating, fire-resistance, naval fires, cork, sustainable fillers

## Abstract

This study explores the potential of natural and recycled materials to enhance the fire behavior of eco-friendly intumescent coatings, compared to a traditional ammonium polyphosphate (APP)-based one. To achieve this, cork, halloysite clay, and recycled glass were evaluated as natural fillers and sustainable components within the coating formulation. The aim was to reduce the reliance on synthetic materials and minimize the environmental impact while maintaining fire performance. Fire exposure tests were conducted to assess the in situ char formation and its relationship to the heat source and char foaming process. The results highlighted that all functionalized coatings exhibited suitable intumescent behavior. The best results were evidenced by cork-filled coating that evidenced an intumescent capacity about 40% higher than the traditional ammonium polyphosphate (APP)-based one. This provided valuable insights into the coating’s real-time response to fire, determining its suitability for various fire-resistant applications.

## 1. Introduction

The study of intumescent coatings’ growth based on fire-retardant coatings is an important area of research achieving a growing interest in several industrial fields, such as naval applications.

Intumescent coatings are functional materials that passively increase a structure’s resistance to fire. Intumescent coatings can withstand high temperatures (usually in the range of 200 °C to 600 °C) for a longer period than conventional coatings [1]. The coatings are commonly used to protect steel substrates exposed to fire, such as structural steel in shipbuilding, where fire-resistance is a critical prerogative with the purpose to increase the structural and health safety [2,3].

Intumescent coatings have been widely used in the naval industry to improve the fire-resistance of various naval structures, including vessels, bulkheads, decks, and engine rooms, to enhance fire safety and in compliance with safety regulations. Naval vessels are often subjected to harsh environments that pose a significant fire risk, and intumescent coatings offer an effective way to reduce this risk, preventing structural collapse and protecting the occupants. Continuous advancements in materials technology are necessary; therefore, intumescent coatings are an exciting research topic aimed at meeting the different and stringent requirements of naval applications.

The driving mechanism that characterizes intumescent coatings is based on a reaction when exposed to heat or flames, forming a multi-layer of foam-like char, which acts as an insulating barrier to protect the underlying material from the fire. This expansion is caused by the release of gases from the coating’s components, such as acid sources and carbonizing agents and foaming agents that collaborate to generate a carbon-rich layer when exposed to heat or flames [4,5]. This char formation creates a safeguarding layer that shields the underlying structure from heat and flames, thereby retarding fire. This porous carbon-rich layer acts also as a barrier layer, slowing down heat transfer to the underlying structure and thereby diminishing the risk of ignition and curbing the spread of fire [6,7]. As reported by Konstantinova et al. [8], polymer thermosets modified with phosphazenes, during the combustion process, release abundant soot and a porous and lacy coke cap is formed on the sample. Thanks to the porous, durable coke cap on the surface of the polymer, heat transfer from the flame into the depth of the sample is reduced.

In this concern, ammonium polyphosphate (APP) is a commonly used acid source in intumescent coatings [9]. Other acid sources include boric acid and diammonium phosphate, while carbonizing agents include pentaerythritol, starch, and mannitol [10,11]. Foaming agents include melamine, urea, expandable graphite, and dicyandiamide [12].

The growth of intumescent coatings is influenced by several factors, including the thermal conductivity of intumescent chars [13], carbon additives [14], or the onset of swelling [15].

Designing a functional intumescent coating that effectively utilizes intumescent agents is highly challenging in this context, as it aims to improve and expand the application contexts of these materials. Extensive research has been conducted on the development and assessment of effective intumescent coatings as a thermal barrier to structural elements and materials, driven by the widespread adoption of intumescent coatings characterized by sustainable components for fire-safe steel structure design [7].

In the last few years, even more researchers have been interested in using sustainable fillers in flame-retardant design [16,17]. This evolution can be attributed to the growing focus on sustainable and eco-friendly methods, especially with regard to intumescent coating fire performance. Scientists want to improve flame-retardant materials’ environmentally friendly impact and effectiveness by combining the above concepts.

Research in the field of sustainable coatings aims to address environmental concerns while maximizing performance. By utilizing sustainable filler as a source able to promote the intumescent performances of the coatings, researchers are exploring innovative alternatives to conventional chemical compounds [18]. As examples, fillers such as coconut fiber [19], wood ash [20,21], coffee husk [22], cocoa shell [23], and eggshell [24] have been explored. These unconventional sources offer the potential to create eco-friendly coatings that maintain or exceed traditional standards for steel fire protection. With continued investigation, these developments hold promise for a more sustainable and efficient coating industry.

With this in mind, in this paper three unconventional sustainable fillers were exerted as potential effective components for green intumescent coatings. The objective of the study is limited to the evaluation of the surface behavior of the material exposed to a flame and not to identify a fire-reaction class for the coating. For instance, cork is a renewable material harvested from the bark of cork oak trees, making it an environmentally friendly choice for fire-retardant coatings. Additionally, nanoclay and glass waste fillers offer the potential to enhance fire safety in coatings through their unique chemical and physical properties. By exploring the incorporation of these sustainable materials into coating formulations, the industry can move towards more eco-friendly and effective fire protection solutions.

To the best of our knowledge, not much research activity has been performed on using these fillers to prevent fire in polymer-based coatings, and their contribution to fire protection is yet to be explored.

A key consideration when incorporating recycled glass into the intumescent coating formulation is the potential impact on the material’s fire-resistant capabilities [25]. Furthermore, the cost implications and overall sustainability benefits of utilizing recycled glass in intumescent coatings warrant careful analysis to determine the economic and environmental feasibility of this approach [26,27]. Incorporating recycled glass into intumescent coatings has the potential to contribute positively to the industry’s efforts to promote environmental sustainability while maintaining high performance standards.

The halloysite nanotubes could beneficially favor, also at low concentration thresholds, the thermal stability and flame-retardancy of the intumescent coating [28]. This can be attributed to the unique structure of halloysite nanotubes, which provide a physical barrier to heat transfer and act as a reservoir for flame-retardant compounds [29,30]. Additionally, the incorporation of halloysite nanotubes in intumescent coatings can lead to improved char formation and reduced smoke release during a fire, thereby enhancing the overall fire safety performance of coated materials [31,32].

Analogously, cork burns without a flame and does not emit toxic gases. Cork powder is characterized by a more effective fire-resistance than other conventional additives, thus slowing down combustion [33] and improving the thermal protective performance of intumescent materials [34]. Moreover, the involvement of all of these options as fire-resistant additives offers the advantage to use natural, environmentally friendly materials, making them an attractive option for various industrial applications.

The aim of this approach was to investigate the potential of these materials, compared to APP, to increase the fire-resistance of the coating, while also contributing to the eco-friendliness of the product. To enhance the sustainability of the product, an alternative approach was proposed, which involved evaluating the intumescent action of cork or halloysite as a natural filler and recycled glass as a sustainable material in the coating under examination. This alternative solution was suggested to reduce the reliance on synthetic materials and minimize the environmental impact of the coating, while maintaining its intumescence performance requirements. The use of cork, halloysite, and recycled glass in the coating could also promote the concept of circular economy and sustainable production practices.

With this in mind, fire exposition tests were performed to assess the in situ char layer growth during the test and to relate a relationship between heat source, char foaming process and intumescent compounds. This testing method provided valuable information on the coatings’ ability to withstand and respond to fire over time, making it an important tool for determining their suitability for use in various applications.

## 2. Materials and Methods

### 2.1. Materials

The coating under consideration is a novel formulation produced by Colorificio Atria S.r.l. (based in Partanna (TP), Italy). The beginning ingredient was an acrylic intumescent coating (termed iC), where ammonium polyphosphate (provided by Budenheim KG, Budenheim, Germany) was added to improve its intumescent characteristics. In an endeavor to simultaneously enhance the intumescent properties and promote the sustainability of the product, three distinct fillers were subjected to thorough investigation. Cork was supplied by Syfar Srl (Acquedolci, Messina, Italy). Commercial halloysite nanotubes (acquired from Sigma Aldrich, Saint Louis, MO, USA) were characterized by a diameter of 30–70 nm and a length of 1.3 μm. Recycled glass was provided by Sarco Srl (Marsala (TP), Italy). The fillers were added using a weight percentage of 20%, 30%, and 1%, respectively, for recycled glass powder, cork powder, and halloysite nanotubes, respecting the workability and applicability of the paint. These fillers were explored as viable alternatives for the coating formulation, as will be discussed in detail later. Structural characterization was conducted by means of X-ray diffraction (XRD, Bruker D8 Advance, Bruker, Billerica, MA, USA), operating in Bragg–Brentano θ–2θ configuration, with CuKα radiation (40 V, 40 mA). XRD patterns were collected in the range 10°–80° with a step of 0.1°/s. Environmental scanning electron microscopy (SEM, FEI Quanta 450, Thermo Fisher Scientific, Waltham, MA, USA) was used for morphological investigation in a low vacuum. Along with the morphological tests, chemical analysis was performed using energy-dispersive X-ray spectroscopy (EDAX, Ametek, Tokyo, Japan) at a 20 kV acceleration voltage.

### 2.2. Coatings Preparation

The samples’ coding and related characteristics are listed in Table 1.

The formulations were applied onto ASTM A1008 carbon steel plates 140 mm × 70 mm × 0.8 mm in size, with an average thickness of 76 ± 10 µm, by a multi-thickness square applicator 2”, 5–50 mils (BYK-Chemie GmbH, Wesel, Germany). Additionally, an uncoated steel plate (uC batch) was used as a reference to better assess the intumescent and fire protection properties of the coatings. Structural characterization was performed by previously described XRD and Fourier Transform–Infrared Spectroscopy (FT-IR) analyses. Infrared spectra were recorded by using a Spectrum Two Perkin-Elmer FT-IR Spectrophotometer (Perkin-Elmer, Waltham, MA, USA). In addition, morphological and thermal characterization by means of the previously described scanning electron microscope and thermo-gravimetric analysis (TGA) was conducted. TGA measurements were performed in air (100 mL/min) in the temperature range 30–1000 °C (scan rate: 10 °C/min), using a TA Instruments SDTQ 600 (New Castle, DE, USA) (balance sensitivity: 0.1 µg).

### 2.3. Fire-Test Equipment

**Figure 1:**
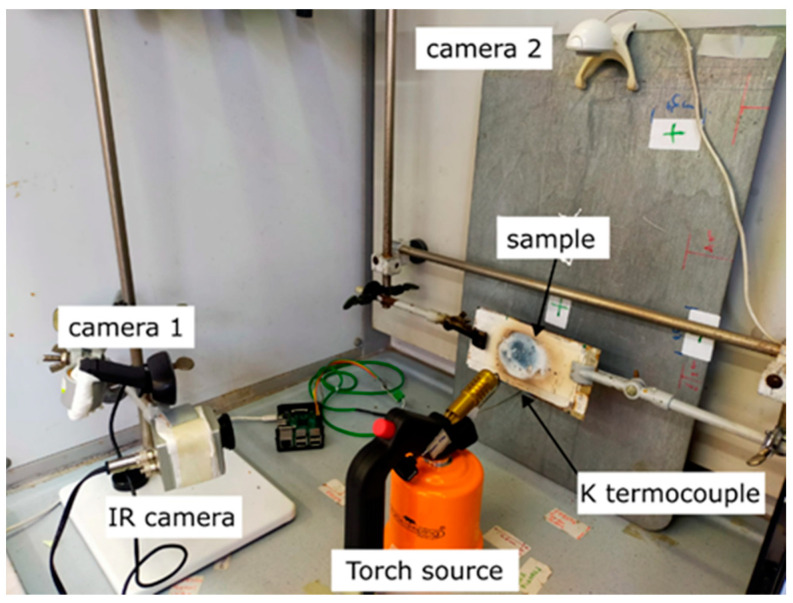
Fire test set-up.

A specific fire-resistance test was performed to assess the behavior under direct flame of the realized functional coatings (the set-up of the test is reported in Figure 1). During the test, the samples were subjected to a direct propane gas torch flame (flame temperature 1980 °C) at a distance of 1 cm from the specimen (300 kW/m^2^) for a roughly 70 s time interval. The heat flow was calculated considering the propane calorific value (14 kW/g·h) and propane consumption, measuring the gas can weight before and after the test, over the investigated time, as reported in a previous published work [35]. Five measurements were conducted for each coating. The coating modification was progressively recorded using three cameras. A thermo-camera (Optris, Berlin, Germany) was positioned directly in front of the specimen to qualitatively monitor the homogeneous temperature distribution on the top side of the sample. Indeed, a camera (Logitech, Lausanne, Switzerland) was located laterally to record the coating profile evolution along its cross-section during the test. To further examine the fire-response capabilities of the coatings and to evaluate the coating’s thermal insulation, the temperature evolution in the back side of the steel plate during the fire-resistance test was recorded by using a K-thermocouple probe placed in contact with the central area of the back side of the substrate. The thermocouple sensor was connected to a data logger to record the temperature data during the test with a frequency of 1 Hz. A complete assessment of the fire-response qualities of the coatings was undertaken by examining the data acquired from these diverse sources.

## 3. Results and Discussion

### 3.1. Sustainable Fillers

**Figure 2:**
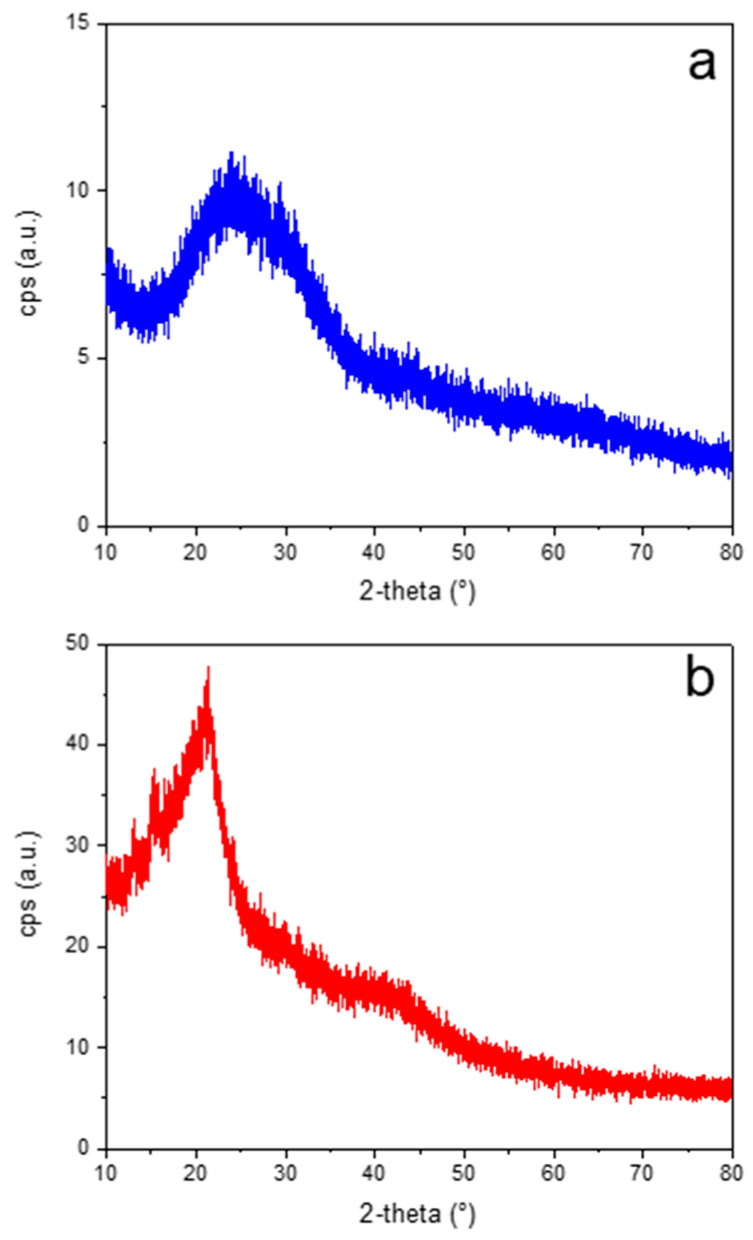

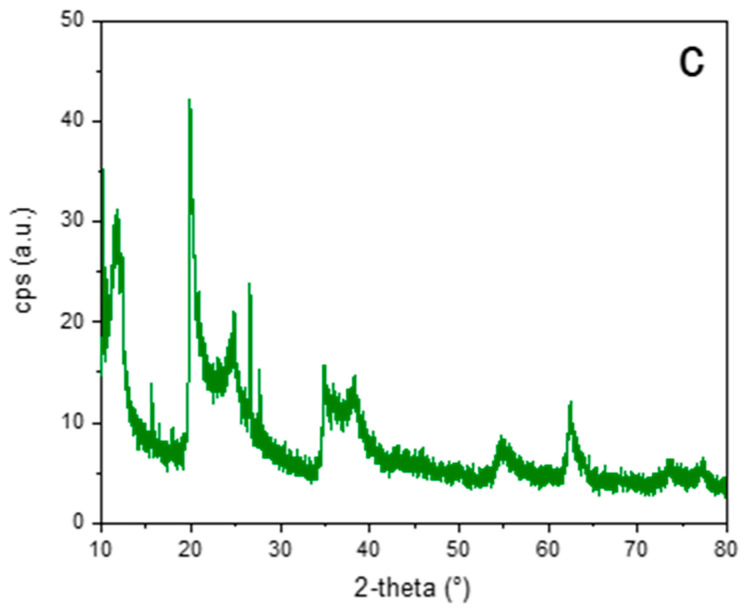
XRD spectra of sustainable fillers: (**a**) recycled glass powder; (**b**) cork powder; (**c**) halloysite nanotubes.

In Figure 2, the XRD analyses of sustainable fillers are reported. As evidenced, in the case of recycled glass powder (Figure 2a), an amorphous diffractogram is obtained. Since glasses do not have a repeating lattice arrangement like crystalline materials, they do not produce distinct diffraction peaks. A similar behavior is observed for cork powder (Figure 2b). Cork is primarily composed of suberin, lignin, and cellulose. Its diffractogram is broad and diffuse, with no sharp peaks associated with crystalline materials. The diffraction pattern shows some broad humps, but these are indicative of the amorphous nature of the cork rather than a specific crystal structure. For halloysite nanotubes (Figure 2c), halloysite peaks (2-theta: 11.87°, 19.97°, 24.78°, PDF-ICDD: 00-009-0453, 2-theta: 19.97°, 26.59°, 62.49°, PDF-ICDD: 00-058-2031) are present. No other structures are observed.

**Figure 3:**
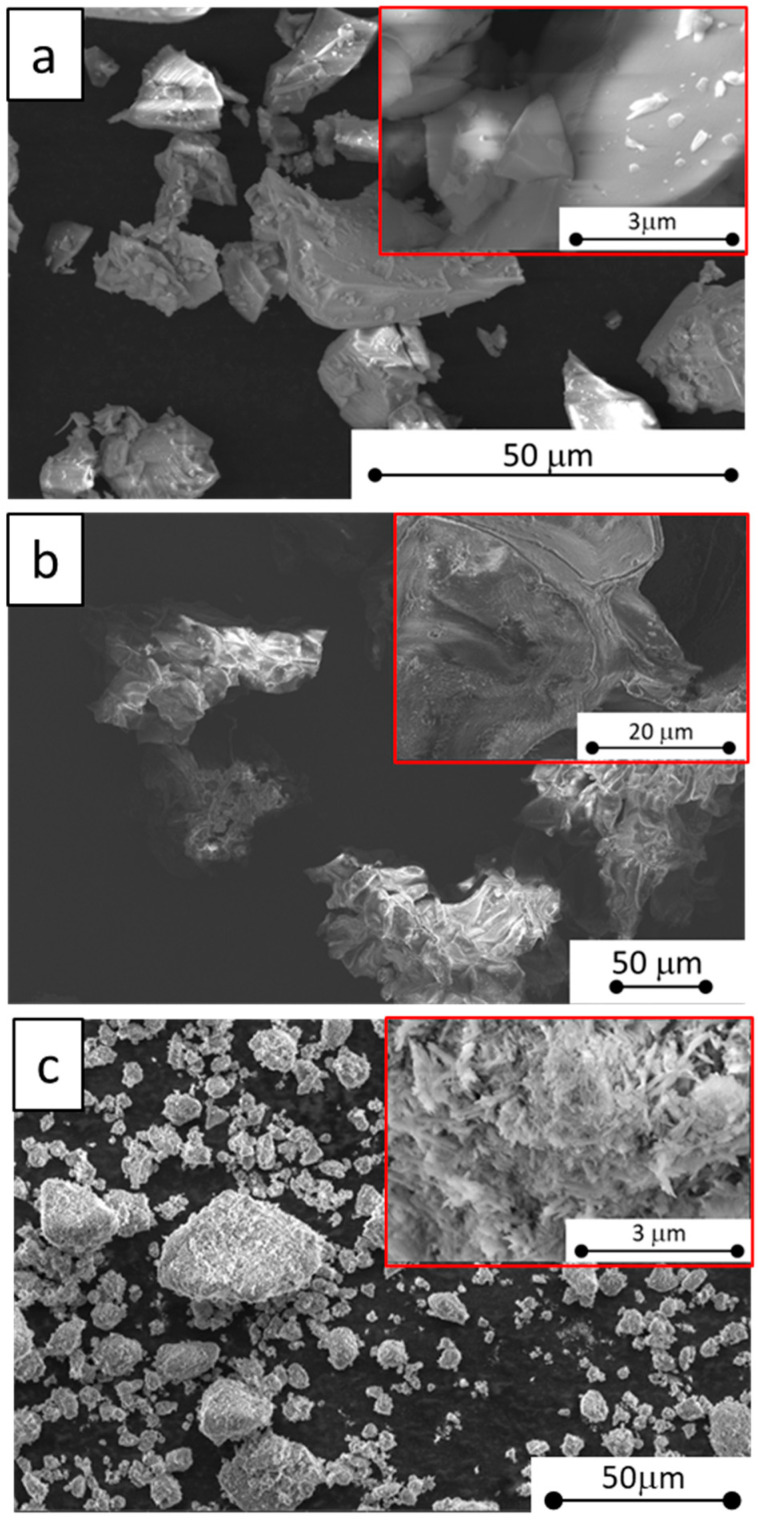
Morphological images of sustainable fillers: (**a**) recycled glass powder; (**b**) cork powder; (**c**) halloysite nanotubes.

By examining the morphology (shape, structure, and size) of the identified eco-friendly components used as additive fillers in commercial iC coating, valuable information is obtained to further enhance its fire-retardant properties. With this in mind, Figure 3 presents microscopic images of these three fillers, allowing us to visually analyze their characteristics and their potential impact on the coating’s performance.

In Figure 3a, referring to the recycled glass powder, it can be observed that the filler exhibits a morphology that is both non-regular and heterogeneous. This means that the shape and the structure of the particles are irregular and differ from each another. More specifically, the size of the particles ranges from just a few microns to approximately 100 µm in diameter.

Figure 3b pertains to the cork powder filler. This filler is characterized by a very significant size. Measurements estimate individual particles to range between 100 and 400 μm, vastly exceeding the dimensions of its glass powder counterpart. This significantly larger size is further visualized by the surface morphology, showcasing a closed-cell structure inherent to cork. The surface is characterized by the presence of flakes, attributable to the walls of the closed cork cell. A magnified detail inset in the top right corner of Figure 3b allows for a closer look at this distinctive feature.

Finally, Figure 3c presents the halloysite nanotubes, showcasing their radically different morphology. Instead of individual flakes, these fillers manifest as microscopic agglomerations, clumping together to form larger particles spanning sizes from a few microns up to 50 µm. Zooming in on the inset detail on the right corner reveals the defining characteristic of these fillers: their tubular structure. Each individual nanotube boasts a high aspect ratio. Measurements indicate diameters hovering between 30 and 100 nanometers, while lengths stretch from 0.3 to 1 µm.

This comparison emphasizes the unique properties of each material, potentially foreshadowing differing roles or performance within the context of the research.

### 3.2. Morphological and Thermogravimetric Analysis of the Intumescent Coatings

**Figure 4:**
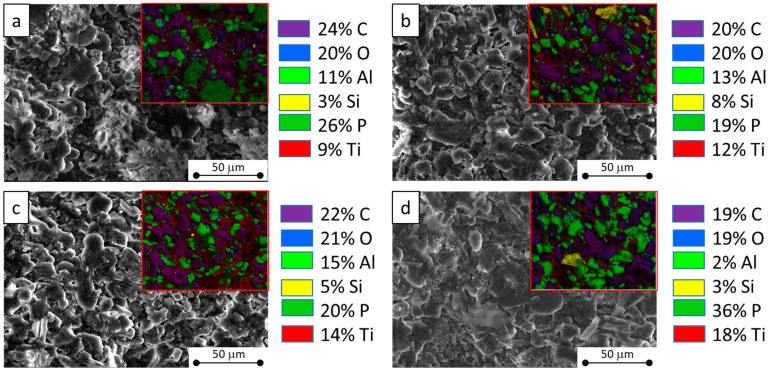
SEM images of the investigated formulations of (**a**) blank coating (iC sample) and (**b**) recycled glass powder (GP-iC sample)-, (**c**) halloysite nanotubes (AN-iC sample)-, and (**d**) cork powder (CP-iC sample)-based composite coatings. The insets represent the EDX mapping of each coating.

With the purpose of providing an initial assessment of the morphological characteristics, Figure 4 showcases scanning electron microscope (SEM) images of all the fabricated APP-based composite coatings.

The SEM images at low magnification reveal a generally similar morphology across all samples, exhibiting a regular texture. Interestingly, all coatings appear to share a common dense and well-packed structural texture with an arrangement of regularly and randomly dispersed particles throughout the matrix. While the cork-based coating has a rougher texture compared to its counterparts using glass and halloysite nanotubes, it still boasts a quite uniform structure with no defects or cavities. Microscopic examination also confirms a crack-free surface indicating that the added particles bind properly with the acrylic binder, demonstrating suitable internal cohesion. The EDX mapping results (insets in Figure 4) highlight a good distribution of the components within the individual coatings. The presence of carbon (in purple) primarily originates from the acrylic matrix and also from melamine and pentaerythritol. Titanium (in red) is added in the form of TiO_2_ that can enhance the thermal and fire stability and the char microstructure of the intumescent coating [35]. Oxygen (in blue) derives from oxides present in the formulation. The green color, representative of phosphorus and aluminum, is present in all the samples, due to the presence of aluminum polyphosphate. There is a greater presence of yellow in the sample added with glass powder and halloysite nanotubes, justified by the addition of silica and nanoclay, respectively. In addition, aluminum is slightly higher in the AN-iC sample due to the structure of the nanotubes filler. This uniformity, coupled with the dense and well-packed texture of the overall sample, hints at an effective structural integrity, making these coatings valid for their potential application as fire-resistant coatings.

**Figure 5:**
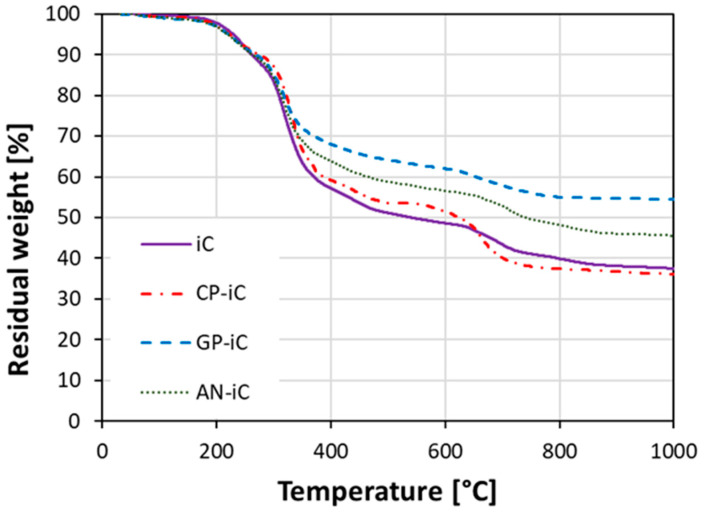
Thermogravimetric analysis of blank coating (iC sample) and recycled glass powder (GP-iC sample)-, halloysite nanotubes (AN-iC sample)-, and cork powder (CP-iC sample)-based composite coatings.

The thermal degradation of composite coatings was assessed by thermogravimetric analysis—TGA (Figure 5). At low temperatures, in the range 30–250 °C, the TGA curves of all coatings exhibited quite a similar trend. The thermal degradation of the coatings was very limited and the residual weight at 250 °C was about 90 wt.%. At increasing temperature above 250 °C, some discrepancies among the batches can be identified.

The GP-iC and AN-iC coatings showed the lowest weight loss, identifiable by the high residual weight evidenced at the end of the test at 1000 °C. The high residue weights of these batches (54.1 wt.% and 45.4 wt.%, respectively) implied that the addition of recycled glass powder and halloysite nanotubes, respectively, enhance the thermal stability and anti-oxidation properties of the coating. This behavior can be justified by the intrinsically relevant thermal stability of the added functional filler that increases the content of high-temperature-stable constituents in the coating formulation.

On the other hand, compared to the refence iC batch, the CP-iC batch (constituted by the addition of cork powder as an intumescent filler) exhibited the highest weight loss at 1000 °C (63.0 wt.% and 64.5 wt.%, for the iC and CP-iC batches, respectively). This behavior can be ascribed to the thermal resistance of cork powder up to intermediate temperatures (T < 550 °C), where a partial degradation of the cork filler takes place, in a range of temperature compatible with the thermal degradation of the iC batch. Hence, the residual weight trend versus temperature of the CP-iC and IC batches is almost compatible in the whole range of investigated temperatures, indicating that cork filler did not induce a modification in the thermal stability of the reference intumescent coating.

### 3.3. Fire Test of the Intumescent Coatings

**Figure 6:**
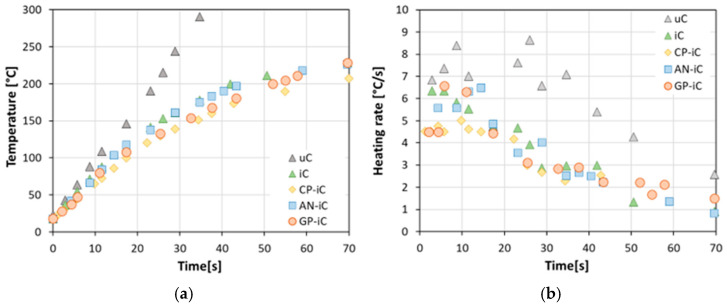
(**a**) Back-side temperature and (**b**) heating rate evolution during fire tests of reference samples of uncoated steel substrate (uC sample), blank APP coating (iC sample), and recycled glass powder (GP-iC sample)-, halloysite nanotubes (AN-iC sample)-, and cork powder (CP-iC sample)-based composite coatings.

Figure 6 shows the back-side temperature evolution during fire test for all the intumescent coatings. Error bars are not reported in Figure 6 to maintain plot readability. Furthermore, summary information regarding these results is summarized in Table 2. Based on the results reported in Figure 6a, the uncoated steel support, as attended, already exhibited a relevant increase in temperature at a low exposure time to the direct flame. After 15 s of exposition, a temperature of ~130 °C was reached on the back side of the steel plate. At the end of the fire test, the uC sample showed the maximum temperature, equal to 389.7 °C (not displayed in the plot for graphical reasons), confirming the low barrier action supplied by the support. A different behavior can be identified for all samples painted with an intumescent coating. The temperature trend for these batches is qualitatively quite similar for all of them, although some discrepancies are identifiable on the evolution of the curve during time.

All samples exhibit a monotone growing trend with increasing time. The iC specimen shows a progressively higher temperature increase compared to the other specimens, indicating that the addition of the sustainable fillers induced a beneficial thermal shielding effect on the insulation performance of the standard intumescent coating. The specimen made with cork powder (CP-iC) is characterized by a temperature on the back side of the panel that is always slightly lower than the other batches. Furthermore, analyzing the performance of the GP-iC specimen in more detail, it is noted that it shows an insulation behavior at low times of exposure to the flame comparable to CP-iC. However, at longer exposure times (time > 45 s), there is a progressive deviation with the temperature that increases until reaching those found for the iC and AN-iC batches. In fact, after 70 s, the CP-iC sample shows a temperature of 207.2 °C, about 20 °C lower than the other samples (Table 2).

In order to better highlight the differences in the evolution of the temperature over time, it is useful to assess the trend of the heating rate, as shown in Figure 6b (calculated as the slope of the temperature over time trend, as shown in Figure 6a).

The heating rate of the uncoated substrate, the uC sample, is significantly higher than the coated samples. The batch with cork powder (CP-iC) shows the best thermal stability, with usually low heating rate values during the overall resistance test fire. The iC specimen, not functionalized with filler, exhibits a non-optimal initial thermal insulation as evidenced by the heating rate of 9.5 °C/s. Furthermore, Figure 6b shows that the intumescent coating functionalized with glass fibers has a relatively effective heating rate curve at low exposure times. As observed in Figure 6a, for time > 45 s, the heating rate of the GP-iC specimen becomes relatively high, indicating a potential degradation phenomenon which reduces the thermal insulation of the intumescent coating.

The drop in the heating rate for all coated samples can be related to the formation of a porous structure in the carbonaceous layer generated during the intumescent process. This behavior is also speeded up by the release of hot gases trapped inside the layer generated during the foaming process of the char [22].

**Figure 7:**
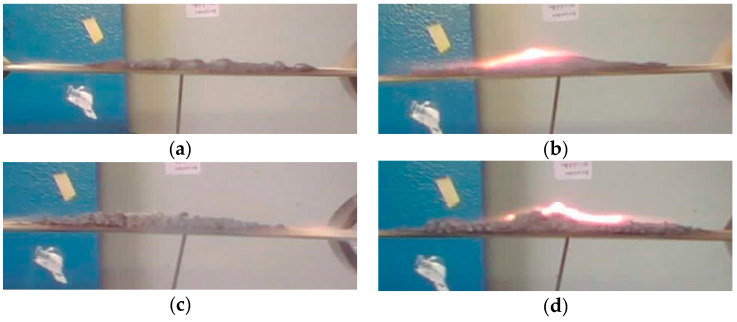
Transverse profile at the end of the fire test (after 70 s of exposition to direct flame) for reference samples of (**a**) blank coating (iC sample), and (**b**) recycled glass powder (GP-iC sample)-, (**c**) halloysite nanotubes (AN-iC sample)-, and (**d**) cork powder (CP-iC sample)-based composite coatings.

With this in mind, Figure 7 compares the transverse profile of the coating at the end of the fire test (after 70 s of exposition to direct flame). The shape of the carbonaceous char is an indirect index of the greater or lesser effectiveness of the proposed intumescent coating. Further details of the intumescent mechanism can be found in [35].

The porous carbon layer is relevant for the GP-iC and CP-iC batches. Both specimens show a comparable intumescence phenomenon. Instead, the batch modified with halloysite shows a limited formation of a carbonaceous layer, very comparable to the reference intumescent coating.

**Figure 8:**
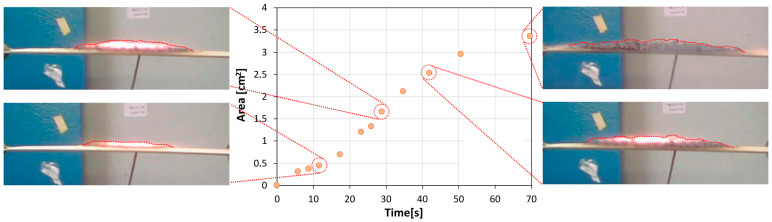
Evolution of the carbonaceous layer area at increasing time with related cross-section images for the GP-iC sample.

In order to better correlate the evolution of the intumescence properties during the fire test of the different coatings functionalized with various sustainable fillers, the swelling profiles of all coatings during the test were recorded. Through digital image analysis, performed using a script appropriately created in Python 3.8, it was possible to quantify the geometric parameters (i.e., shape, width, height, and area) of the porous char layer formed progressively over time during the fire test. The results of the cross-sectional area of the char layer generated for the glass waste filled coating (GP-iC batch) with the associated digitized images at varying times are shown in Figure 8.

The evolution of the porous char layer area has a monotonically increasing trend. At approximately 20 s of exposure to fire, the curve shows a knee which indicates the relevant activation of the intumescence phenomena which increase the area enflamed by the swelling and foaming process of the char. The cross-section images of the sample in this range of time confirm the abrupt increase in the width and thickness of the intumescent area. This phase undergoes a slight slowdown only at exposure times close to 60 s for which there is a reduction in the slope of the curve. This is confirmed by evaluating the cross-section images at long exposure time that exhibited a quite similar shape, pointing out the almost completed intumescent process of the GP-iC sample.

**Figure 9:**
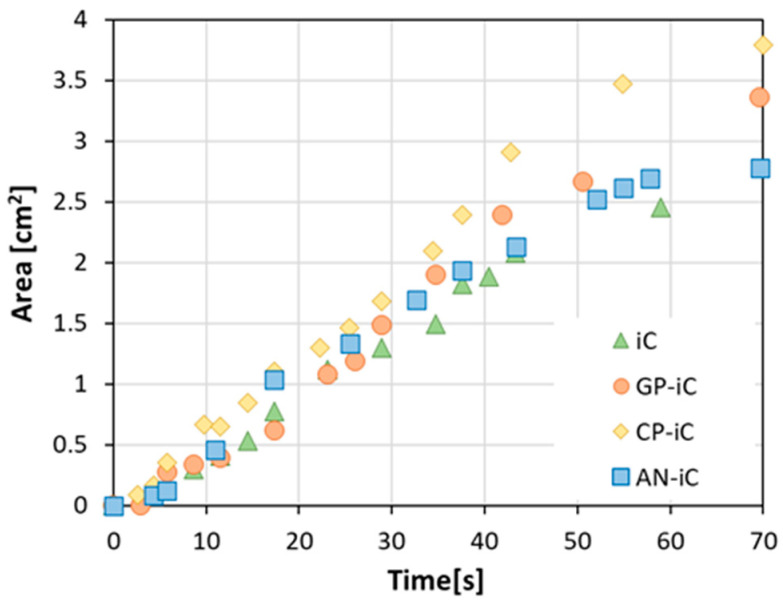
Cross-section intumescent area during fire tests of blank coating (iC sample) and recycled glass powder (GP-iC sample)-, halloysite nanotubes (AN-iC sample)-, and cork powder (CP-iC sample)-based composite coatings.

Figure 9 compares the results of the cross-section intumescent area for all of the coated batches. The more the curve slopes upwards, the greater the intumescence effect of the coating.

At low flame exposure times, all specimens exhibit comparable intumescence phenomena. The intumescence area curves demonstrate a remarkably similar trend among the different batches. However, a distinct difference in behavior is observed at long exposure times. Specifically, the iC batch rapidly exhibits stabilization of the area. This can be attributed to the saturation of the intumescence process and the resulting stabilization of the shape and size of the porous char layer formed during the fire test. Similar considerations can be applied to the AN-iC batch, although it is characterized by a 13% higher intumescence effect compared to the blank batch (iC). Conversely, the CP-iC and GP-iC batches demonstrate an improvement in intumescence properties, with the former more effective than the latter. After approximately 35 s, the trend of the area curve underwent an abrupt increase associated with the addition of cork or glass, respectively, which amplified the protective action of the coating against fire. After 70 s, the cross-section intumescent area in the CP-iC and GP-iC batches was 3.79 cm^2^ and 3.36 cm^2^, respectively. These results indicate that the best outcomes were evidenced by cork-filled coating that evidenced an intumescent capacity about 40% higher than the traditional ammonium polyphosphate (APP)-based one. In the literature, various polymeric materials implemented with intumescent additives have been studied, as already mentioned in Section 1. The uniqueness of the present work lies particularly in the fact that the subject of study is a thin coating, while most studies investigate materials with significantly greater thicknesses. Furthermore, if we compare this study, for example, with the work by Isitman et al. [36], where carbon nanotubes are used in organophosphorus flame-retardant poly(methyl methacrylate), the additives used here are green and sustainable, as they are recycled materials.

This unique feature makes cork powder an excellent choice for enhancing the fire-resistance of various materials, including paints and coatings. Additionally, cork powder is a sustainable and environmentally friendly material, making it an attractive option for companies looking to improve the safety and sustainability of their products. As the demand for fire-resistant materials continues to grow, the use of cork powder as an additive is expected to become increasingly prevalent in a wide range of industries. Its ability to effectively slow down the combustion process and enhance thermal protective performance makes it an invaluable component in the development of safer and more resilient products for both consumer and industrial applications. Moreover, it could use recycled cork, reducing waste and promote sustainability by giving new life to already exploited materials.

## 4. Conclusions

In pursuit of safer and greener fire protection, this study explored the potential of novel intumescent coatings infused with sustainable fillers. To achieve this, cork, halloysite clay, and recycled glass were evaluated as natural fillers and sustainable components within the coating formulation. These eco-friendly alternatives were tested against a conventional coating (iC) to evaluate their fire-resistance.

The results demonstrate that all samples exhibit a growing trend in temperature over time, with the iC specimen showing the highest temperature increase, indicating the positive influence of sustainable fillers on insulation.

Among the tested variations, the cork-filled (CP-iC) specimen stands out for its superior performance. It exhibits a significantly lower temperature on the back side compared to other samples, reaching a maximum of 207.2 °C, which is 20 °C lower than the iC sample after 70 s of exposure.

Digital image analysis confirms the enhanced intumescent behavior of the CP-iC specimen. The porous char layer area grows steadily with increasing fire exposure, with rapid expansion occurring around 20 s, due to char swelling and foaming. Expansion slows near 60 s, as the intumescent process in the coating approaches completion.

All samples behaved similarly at short fire exposure. At longer exposure, the cork-filled (CP-iC) coating outperformed the other products, showing a 40% higher intumescent capacity than the traditional (iC) one. This improvement is attributed to the addition of cork, amplifying the fire protection.

While traditional intumescent coatings have established effectiveness in fire protection, cork-filled coatings represent a promising alternative with distinct advantages in thermal insulation, cost, and sustainability. The use of cork not only respects fire-retardancy but also aligns with the growing demand for environmentally friendly materials. The results obtained in this study represent a starting point for the development of innovative formulations. Further investigations will be conducted to extend the results obtained to thicker intumescent systems, with the aim of optimizing formulations for specific applications.

Overall, this study highlights the promising potential of sustainable fillers like cork in improving the fire-resistance of intumescent coatings. The superior performance of the CP-iC specimen suggests its potential for applications requiring enhanced fire protection for naval fire safety.

## Figures and Tables

**Table 1:** Samples coding and related characteristics.

Code	Characteristics
iC	Intumescent coating
GP-iC	Intumescent coating enhanced with recycled glass powder
AN-iC	Intumescent coating enhanced with halloysite nanotubes
CP-iC	Intumescent coating enhanced with cork powder
uC	uncoated

**Table 2:** Main summary data from temperature evolution during fire-resistance tests of the coatings.

Code	Max Heating Rate (°C/s)	Max Temperature (°C)
iC	6.3 ± 0.8	228.8 ± 25.4
GP-iC	6.6 ± 0.7	228.7 ± 23.6
AN-iC	6.5 ± 0.8	226.4 ± 26.8
CP-iC	5.0 ± 0.6	207.2 ± 22.9
uC	8.7 ± 0.9	389.7 ± 31.2

## Data Availability

The original contributions presented in the study are included in the article, further inquiries can be directed to the corresponding author.
